# SATS: simplification aware text summarization of scientific documents

**DOI:** 10.3389/frai.2024.1375419

**Published:** 2024-07-10

**Authors:** Farooq Zaman, Faisal Kamiran, Matthew Shardlow, Saeed-Ul Hassan, Asim Karim, Naif Radi Aljohani

**Affiliations:** ^1^Scientometrics Lab, Information Technology University, Lahore, Pakistan; ^2^Department of Computing and Mathematics, Manchester Metropolitan University, Manchester, United Kingdom; ^3^Department of Computer Science, Syed Babar Ali School of Science and Engineering (SBASSE), Lahore University of Management Sciences, Lahore, Pakistan; ^4^Information Systems Department, Faculty of Computing and Information Technology, King Abdulaziz University, Jeddah, Saudi Arabia

**Keywords:** scientific documents, summarization, simplification, transformer model, deep learning

## Abstract

Simplifying summaries of scholarly publications has been a popular method for conveying scientific discoveries to a broader audience. While text summarization aims to shorten long documents, simplification seeks to reduce the complexity of a document. To accomplish these tasks collectively, there is a need to develop machine learning methods to shorten and simplify longer texts. This study presents a new Simplification Aware Text Summarization model (SATS) based on future n-gram prediction. The proposed SATS model extends ProphetNet, a text summarization model, by enhancing the objective function using a word frequency lexicon for simplification tasks. We have evaluated the performance of SATS on a recently published text summarization and simplification corpus consisting of 5,400 scientific article pairs. Our results in terms of automatic evaluation demonstrate that SATS outperforms state-of-the-art models for simplification, summarization, and joint simplification-summarization across two datasets on ROUGE, SARI, and **CSS_1_**. We also provide human evaluation of summaries generated by the SATS model. We evaluated 100 summaries from eight annotators for grammar, coherence, consistency, fluency, and simplicity. The average human judgment for all evaluated dimensions lies between 4.0 and 4.5 on a scale from 1 to 5 where 1 means low and 5 means high.

## 1 Introduction

The automated generation of simplified summaries of scholarly articles is a popular mechanism for the public dissemination of scientific discoveries (Hou et al., 2022). This task can be performed by leveraging text summarization (Tomer and Kumar, 2022) and text simplification (Al-Thanyyan and Azmi, 2021), which are well-defined tasks in Natural Language Processing (NLP) (Iqbal et al., 2021). Text summarization is the task of reducing text size while maintaining the information presented in the text. Using text summarization, a long text is provided as input and by automated means a shorter and more concise version is generated (Cai et al., 2021). Text simplification reduces the complexity of a text while retaining the original meaning by producing an easy-to-read version of the input source text (Shardlow, 2014b; Alva-Manchego et al., 2020b). Text simplification typically operates at the sentence level, with each complex sentence being transformed into a simpler alternative.

At the intersection of text simplification and summarization, lies the combination of these two tasks. The desired output is summarized (shorter in length) and simplified (simple to read and understand). We argue that, if a text is only summarized, technical words may remain, impeding a reader. If it is only simplified, the resulting text might be too long and repetitive. Therefore, having both simplification and summarization is vital for true understanding in heterogeneous areas such as public health literature, legal texts, or scientific communications.

As an example, consider two pairs of texts in Table 1. In source 1 and source 2, there are technical terms such as “locus,” “perturb,” “microbial,” “apex predator,” “juxtacrine axo-glial,” and “myelination.” These technical terms make it difficult for a lay reader to digest; on the other hand, both the summaries are clear, concise, and easy to consume and digest.

**Table 1:** Examples of simple summaries.

**Source 1**	**Single gene locus changes perturb complex microbial communities**
	**as much as apex predator loss**
Summary 1	Genetic mutants alter entire biological communities
Source 2	Spatial mapping of juxtacrine axoglial interactions identifies novel molecules
	in peripheral myelination
Summary 2	New technique lets scientists see and study the interface where two cells touch

The key focus of this study is to model the task of summarization and simplification to be executed simultaneously using deep learning methods (Zerva et al., 2020); for this objective, we have extended the ProphetNet (Qi et al., 2020) architecture to perform simplification and summarization.

The main contributions of this study are as follows: (a) we propose a simplification aware loss function for the joint task of simplification and summarization as explained in Section 3.1; (b) we evaluate the proposed model against two datasets; Eureka (Zaman et al., 2020) and CNN-Daily Mail (See et al., 2017); and (c) we demonstrate that our model outperforms the previous state of the art on combined summarization and simplification.

The rest of the study is organized as follows: Section 2 presents related work on recent summarization, simplification, and evaluation indices. Section 3 presents data and methods, including state-of-the-art models. While Section 4 discusses the results of this study, Section 5 presents concluding remarks.

## 2 Related work

Text summarization is the task of compressing a text by automated means while preserving meaning. Various NLP approaches exist for text summarization (Mao et al., 2021; Suleiman and Awajan, 2022). There are two ways of achieving text summarization: *extractive* and *abstractive*.

### 2.1 Extractive summarization

In extractive text summarization, the essential sections of the original document are first marked, and then those important sections are arranged in a document to produce the summary. The first attempt at text summarization introduced automated ways of producing summaries (or abstracts) of the original documents (Baxendale, 1958; Luhn, 1958). It was found that the count of occurrence determines the importance of a word; hence, the importance of a sentence was obtained by counting the important words in that sentence (Luhn, 1958). It was also found that the location of a sentence in a document determines its importance. It was further explored that in 85% of paragraphs, the important sentence is located at the start of the paragraph and 7% of the paragraphs it is located at the end (Baxendale, 1958). Later, Edmundson (1969) manually wrote the summaries of 400 documents and used the word frequencies from Luhn (1958) and location-based sentence importance from Baxendale (1958) to produce summaries.

A supervised approach has been taken for extractive summarization (Collins et al., 2017), and to extract essential sentences, regression was used as sentence scoring (Zopf et al., 2018).

Extractive text summarization has been extended to multi-document level (Sanchez-Gomez et al., 2020). In this study, the authors explored the Artificial Bee Colony algorithm for extractive summarization, and for feature extraction, TF-IDF approach was tailored. The authors formulate the summarization task as an optimization problem and set two objectives: (1) coverage and (2) redundancy. The coverage covers the main contents; redundancy is concerned with avoiding and controlling the redundant sentence selection in the output summary. Extractive text summarization has also been explored through the lens of query-based sentiment analysis (Sanchez-Gomez et al., 2021). In this study, the authors explored multi-document summarization with query-based and sentiment score-based approaches; that is, the output summary will have an identical sentiment score to the input user query over a set of source documents.

Recently, deep learning has also been studied for extractive (Kinugawa and Tsuruoka, 2017) and abstractive (Liu et al., 2015; Chen and Bansal, 2018) summarization. A study Mackie et al. (2014) revealed that sumBasic algorithm (Nenkova and Vanderwende, 2005) performs well for the task of microblogs summarization. This approach is good for producing long and fluent text passages but produces non-factual text in the output summaries (See et al., 2017). To address this problem, Generative Adversarial Neural Networks (Goodfellow et al., 2014) have been applied (Liu et al., 2018).

The performance of extractive summarization may be greatly improved by identifying structured elements in text (Filatova and Hatzivassiloglou, 2004), across genres such as news articles (Thompson et al., 2017) and medical text (Shardlow et al., 2018).

### 2.2 Abstractive summarization

Abstractive summarization exploits deep learning methods such as sequence-to-sequence models to generate the output summary based on the input text (Liu et al., 2015; Chen and Bansal, 2018). The authors in Azmi and Altmami (2018) extended abstractive summarization with the granularity level of the generated summary to be used and controlled by the end-user. The authors in Mehta and Majumder (2018) studied the aggregation of multiple models for text summarization tasks. In a recent study (Barros et al., 2019), the idea of using multi-document as input for text summarization has been studied, the authors applied text summarization for news articles, the idea used in this study is the narrative approach, events from different news articles were first extracted and then sorted over a timeline, after sorting, and for each event, one-line output has been generated based on the contextual information available in news articles. The authors reported that their proposed narrative approach generates better summaries than other state-of-the-art methods. Abstractive text summarization is also been studied in the biomedical domain (Van Veen et al., 2023; Yang et al., 2023). Large language models (LLM) have been studied for text summarization (Liu et al., 2023), and it was found that the LLM generates very fluent summaries but commits mistakes such as generation of non-factual text in the output summary. Abstractive text summarization has been explored for the Greek Language (Giarelis et al., 2023). More recently, a study Rehman et al. (2023) adp summarization.

The combination of abstractive methods with extractive methods has been explored recently (Cho et al., 2019). Such work leads to selecting some parts from the source text and generating new content for diversification. The authors designed their setup with three decoders to generate three output summaries that are different from each other; for selecting contents from the source text, each chunk of the input text was scored; they call it “focus.” Instead of training both encoder and decoder, utilization of pre-trained encoder such as BERT (Devlin et al., 2019) for the task of summarization both abstractive and extractive has been explored (Liu and Lapata, 2019). In this study, the authors reported that setting a pipeline of the pre-trained encoder and a fine-tuned decoder improves the performance of text summarization methods. As pre-training improves the performance of text summarization methods, in Dong et al. (2019) self-supervised pre-training was explored. The authors used a transformer for summarization with the supervised objective in this study. Pre-training and self-supervised objectives were further explored for the task of summarization (Zhang et al., 2019a), and the authors investigated the use of masking for extractive methods, that is, masking some tokens in the input text and treating them as targets to make the source-target pairs for supervised training. The self-supervised pre-training has been recently explored in Yan et al. (2020) for summarization. The authors used masking; some input tokens are marked as masks and presented as missing. The objective is set to predict those missing tokens, the masked input, and the masked token as target presented to the training model. The authors further explored the prediction/generation of more than one token at a time on the decoder side.

In See et al. (2017), the authors used pointer generator network for text summarization (Vinyals et al., 2015). The output summary of this model is both abstractive and extractive. A mechanism called coverage was proposed to avoid the generation of repetitive tokens in the generated summary.

### 2.3 Text simplification

Automated text simplification is a new problem that lies under the domain of NLP, that is, a text modification process aiming at making the text easy to understand. In Hoard et al. (1992), the authors worked on writing technical manuals and assisting stroke survivors to read (Carroll et al., 1998). In the academic literature, text simplification can be found in three categories: neural, syntactic, and lexical simplification.

In syntactic text simplification, the structure of the grammar is rewritten such that it transforms its constituents such as voices and narration or long sentences into small understandable pieces (Siddharthan, 2014). This approach is different from the lexical approach (Shardlow, 2014b). In the lexical approach, complex words are identified at first; then, their substitutes are generated. After that, the step of word sense disambiguation is performed. Finally, the substitutes are re-ranked, and a selection is performed for the final synonym of the original word. This approach produces the output with significant errors (Shardlow, 2014a).

Neural machine translation models can be modified to generate a hybrid of syntactic and lexical text simplification. In Wubben et al. (2012) and Li et al. (2018) statistical machine translation model was used. Recently, neural machine translation has been applied for text simplification (Nisioi et al., 2017). Further work has shown that the level of complexity of the output can be controlled (Agrawal and Carpuat, 2019; Marchisio et al., 2019; Nishihara et al., 2019). Recent work on simplification has focused on the improvement of datasets (Alva-Manchego et al., 2020a) and evaluation measures (Alva-Manchego et al., 2019). In Macdonald and Siddharthan (2016) simplification was used as a preprocessing step prior to summarization of children's stories. Simplification was also used similarly more recently for clinical data summaries generation (Acharya et al., 2019).

#### 2.3.1 Sentence compression

Sentence compression eliminates repeated information from a sentence while preserving key contents present in the original sentence. The first attempt without using linguistic features for sentence compression was made in 2015 by Katja et al. at Google (Filippova et al., 2015). In this study, the authors proposed an approach based on LSTM for token deletion, which led to omitting the redundant information present in the sentence. The authors tuned and evaluated their proposed LSTM-based method against sentence compression dataset (Filippova and Altun, 2013). The authors found that the simple LSTM-based method produces readable and more informative compression without using any syntactic information. The authors furthermore reported that syntactic information does not contribute to performance. Later, Wang et al. (2017) proposed an extended LSTM-based model which incorporates syntactic information of Part of speech and dependency parsing in the form of embedding, and the authors further imposed minimum and maximum sentence length constrained over the compressed output. Sentence compression methods perform well on short-length sentences (Kamigaito et al., 2018). In recent work, sentence compression has been explored to improve the capability of sentence compression methods for long sentence lengths. The authors used attention-based weights and dependency trees to capture syntactic information. The proposed method is evaluated using token level F1 and Rouge scores (Lin and Och, 2004) and reported that their proposed method outperforms state-of-the-art methods for sentence compression task (Kamigaito et al., 2018). Sentence compression has also been studied in an unsupervised manner (Zhao et al., 2018). In this study, a reinforcement-based approach is used for sentence compression. The authors used a language model as an evaluator; a series of deletion and evaluation passes were executed consecutively, such as first a deletion operation is performed, then an evaluation is performed to assess the correctness of the resultant sentence. The sentence compression task is studied to improve the grammar of the compressed sentences (Kamigaito and Okumura, 2020). In this study, the author enhanced the decoder part of the LSTM-based model with extra information of the parent word and child word from the dependency tree.

#### 2.3.2 Sentence splitting

Sentence splitting is the task of breaking longer sentences into shorter pieces so that each individual piece is a complete sentence; the aim is to reduce the complexity of longer sentences; this comes under text simplification. Sentence splitting can further be used in other NLP tasks such as machine translation. Splitting can also help second language learners to read a longer sentence in small pieces (Narayan et al., 2017). Recently, Aharoni and Goldberg explored that the performance of sentence splitting can be improved by employing a copy mechanism; a copy mechanism is used in other tasks such as abstractive text summarization (See et al., 2017). It was further observed that a unique train-validation-test split is required to validate the performance of sentence splitting (Aharoni and Goldberg, 2018). Aharoni and Goldberg explored the dataset for sentence splitting should have train-validation-test sets to be unique and crafted carefully, they observed that in the previous studies, the dataset splitting was not carefully carried out, they further analyzed that validation and test sets contained more than 89% of unique simpler sentences from the train set, and this was making the sequence-to-sequence models to memorize the simpler sentences and thus leads to high-performance scores. Later, a new corpus for the task of sentence splitting is built (Botha et al., 2018), with this new corpus, machine learning models are able to capture more information present in the corpus than before with the previous benchmark by Narayan et al. (2017), and using this, the models were trained with unique and disjointed train-validation-test sets. The authors established a new state of the art with almost double the previous results.

### 2.4 Evaluation metrics for text simplification and summarization

To quantify the performance of any machine learning, deep learning or NLP task, one or more metrics are used, such as accuracy, precision, recall, and F1 measure. A good metric captures all the critical aspects of a task it is designed to evaluate. For text simplification, a widely used metric known as SARI (Xu et al., 2016) is used, although some metrics such as BLEU are adopted from other NLP tasks such as machine translation (Papineni et al., 2002). For the evaluation of summarization systems, the ROUGE metric (Lin and Hovy, 2003) is used widely; however, due to the limitations of the existing ROUGE metric, it must be addressed carefully (Schluter, 2017). Another evaluation method has been proposed in Jia et al. (2023) which evaluates consistency and faithfulness aspects. In the following subsections, we will discuss some appropriate evaluation metrics.

#### 2.4.1 BLEU

The best way to measure the performance of machine translation tasks is human judgment, but human judgment requires time and effort, which is a time-consuming process. To automate this process, the BLEU metric was introduced (Papineni et al., 2002). The advantages of using BLEU are that it is fast and easy to compute, language-independent, and correlates with human judgements. As BLEU work by matching and counting the n-grams where *n*∈{1, 2, 3, 4} between reference text and candidate text generated by the machine translation system. BLEU does not take word order into account and uses the modified precision with brevity penalty. For full mathematical detail, the reader is referred to the work presented in Papineni et al. (2002). BLEU is also used to measure the performance of many text generations tasks such as abstractive text summarization, automatic image caption generation, and question answering tasks.

#### 2.4.2 ROUGE

ROUGE is a measure used for the evaluation of text summarization systems, and ROUGE stands for Recall-Oriented Understudy for Gisting Evaluation (Lin, 2004). Similar to BLEU (Papineni et al., 2002), the ROUGE metric also measures the overlap between system-generated summaries and the gold standard reference summaries. One difference between ROUGE and BLEU is that BLEU is precision-based where ROUGE is recall-based. Similar to BLEU, ROUGE also used multiple references to match with the generated summary; in case of multiple references, ROUGE measure computes the pairwise score with each reference and then take the maximum of all the pairs.

#### 2.4.3 METEOR

METEOR is a metric used to evaluate the performance of machine translation systems (Banerjee and Lavie, 2005), Meteor is also a unigram matching-based metric, which finds the overlap between the system-generated output summary and the reference ground truth summary, similar to BLEU (Papineni et al., 2002) and ROUGE (Lin, 2004). METEOR is both unigram precision-based and unigram recall-based, similar to BLEU and Rouge. The authors' enhanced capability is that METEOR accounts for the order of the matched unigrams.

#### 2.4.4 SARI

SARI is a text simplification metric (Xu et al., 2016), used to measure performance of text simplification systems. Text simplification systems are based on the three basic approaches: (1) Simplification by splitting longer sentences into their constituent counterparts, (2) Simplification by deletion of complex words and reordering, (3) simplification by paraphrasing and replacing complex words with their simple counterparts (Feng, 2008). To capture the three types of operations discussed earlier, SARI considers the addition of tokens, deletion of tokens, and retention of tokens and aggregates the count of these three operations into one final score. SARI compares the system-generated output with both single or multiple references and the input source text.

#### 2.4.5 SAMSA

SAMSA is a text simplification metric (Sulem et al., 2018), used to evaluate text simplification systems. Unlike other metrics, SAMSA is a reference-less evaluation metric that does not compare the system-generated output with the ground truth references. Unlike SARI (Xu et al., 2016) SAMSA considers the structural aspects of the generated text. It finds scenes in a sentence and then look for a single scene to be present in a single sentence and thus have the mapping between the scenes present in the input and the splits of the scenes in the output.

#### 2.4.6 FKGL

Flesch Kincaid Grade Level (FKGL) is an evaluation metric widely used to measure the readability scores and grade level of the input text (Kincaid et al., 1975). The FKGL metric translates the readability score into a U.S. school grade level, making it easier to understand the reading difficulty of a given text. The formula to compute FKGL score is given in Equation (1).


(1) $$\begin{array}{l} FKGL=0.39\times \frac{N_{words}}{N_{sentences}}+11.8\times \frac{N_{syllables}}{N_{words}}-15.59 \end{array}$$

#### 2.4.7 BERT SCORE

BERTScore is used to evaluate text summarization, machine translation, and other text generation models. Unlike BLEU (Papineni et al., 2002) and ROUGE (Lin, 2004), it computes scores and evaluates model outputs even if there are no lexical matching words but there are semantically matching words in the gold reference text and the generated candidate text (Zhang et al., 2019b). BERTScore uses contextual embedding to compute semantic similarity between reference and generated text. BERTScore is also word order invariant, unlike BLEU it computes semantic similarity between generated text and the gold standard reference without word order. BERTScore has shown high correlation with human judgments.

#### 2.4.8 CSS1

To measure the effectiveness of the hybrid model for text summarization and simplification, a metric has been proposed in Zaman et al. (2020). This metric measures both the summarization and simplification and then takes the F1 measure of both to compute the once single score, which can provide insights for both the aspects (simplification and summarization) of the hybrid models. CSS1 can be formulated using *ROUGE*_1_ and *SARI* score as in Equation (2)


(2) $$\begin{array}{l} CSS_{1}=2\times \frac{R_{1}\times SARI}{R_{1}+SARI} \end{array}$$

### 2.5 Resources for text simplification

Automated text simplification is transforming a complex text into a simpler one that should be easy to understand. This task can be handled in many ways, as discussed in Section 2.3. Different kinds of resources are required to perform text simplification, depending upon the type of simplification, such as lexical (Shardlow, 2014a,b) simplification or structural simplification and the method used such as sequence-to-sequence learning (Liu et al., 2015; Chen and Bansal, 2018), machine translation. Considering the machine-translation method for text simplification (Zhu et al., 2010; Xu et al., 2016) requires a vast amount of parallel monolingual corpus and a considerable number of parameters to be optimized during training. So heavy computational resources are required to accommodate such large corpora in memory alongside the model parameters. For instance, working in the domain of text simplification, one can build and train state-of-the-art models only if rich computational resources are available. The availability of such resources is a big problem that the text simplification community is facing. Developing optimized resource algorithms for text simplification are required to advance the domain's further state of the art. Below are some benchmark datasets used to train text simplification models; further detail is given in subsections.

#### 2.5.1 PWKP corpus/WikiSmall

In 2010, Zhu et al. collected a monolingual parallel corpus containing 108k sentences and its simplified pairs, and this dataset was harvested from around 65k Wikipedia articles and its corresponding simple Wikipedia articles (Zhu et al., 2010). The authors aligned the sentences in 1-to-1 and 1-to-many fashion. In the later alignment, the authors used sentence splitting—the corpus can be found at the link.^1^

#### 2.5.2 Turk corpus/WikiLarge

Xu et al. (2016) explored a statistical machine translation model for text simplification purposes; along with this, they introduced a new metric for text simplification evaluation and a new dataset named Turk corpus; they used some filtered data from PWKP corpus (see section PWKP), along with this modified PWKP, they used multiple 8 reference sentences of each original sentence, and this was done through Amazon Mechanical Turk. There are 2,000 sentences for tuning and 350 sentences for testing; for training the simplification models, most researchers use WikiLarge (Zhang and Lapata, 2017), or WikiSmall dataset (see section 2.5.1).

#### 2.5.3 ASSET corpus

Alva-Manchego et al. (2020a) prepared a new dataset for text simplification task, which is based on Turk corpus (see Section 2.5.2). The authors selected the same source sentences with modified reference sentences. The idea behind maintaining multiple references is that multiple paraphrase operations can simplify. ASSET corpus contains both 1-to-1 and 1-to-many alignments. Unlike Turk corpus, ASSET contains 10 references for each source sentence. The tuning and test set size is the same as Turk corpus that is 2,000 tuning examples and 350 test examples. The ASSET dataset can be downloaded from the link.^2^

### 2.6 Resources for text summarization

Different kinds of resources are required to perform text summarization, depending upon the approach and method employed. Consider the two broad methods of text summarization, Extractive and Abstractive; some Extractive summarization methods only rank parts of the input text and then output the filtered portion of the input text such as text rank (Mihalcea and Tarau, 2004), and such approaches do not require many resources as compared to the Deep Neural Network-based methods such as Kinugawa and Tsuruoka (2017). Similarly, Abstractive summarization methods such as See et al. (2017) and Yan et al. (2020) are based on sequence-to-sequence learning which requires millions of parameters to be tuned; in addition to a vast number of parameters and computational resources, these methods required a large corpus of parallel pairs of input long documents and reference summaries. The requirement of such resources becomes a bottleneck for the research communities. To further advance state of the art in the domain, more advanced algorithms are required that are resource optimized. In the next subsections, we discuss the datasets available for summarization research.

#### 2.6.1 Gigaword

In 2003, Graff et al. compiled a dataset named Gigaword (Graff et al., 2003) that was mainly used for sentence summarization tasks or generation of headlines tasks. The size of the source and target summaries in terms of tokens is short; the statistics of the dataset are as follows: there are 3.8M training pairs, 189k validation pairs, and 1951 test pairs. The model trained and developed with this dataset are primarily evaluated with ROUGE (1, 2, L) scores.

#### 2.6.2 CNN-daily mail dataset

For text summarization research CNN-daily mail prepared by Nallapati et al. (2016) is a large dataset containing 287,226 training article pairs, 13,368 validation article pairs, and 11,490 test article pairs. Researchers tune and validate their developed models with CNN-daily mail dataset and report ROUGE-1, ROUGE-2, and ROUGE-L scores. There are multiple versions available such as the entity-anonymized version (Nallapati et al., 2016) and the non-anonymized version (See et al., 2017). A pre-processed version (See et al., 2017) of the dataset can be downloaded.^3^

#### 2.6.3 X-Sum

Narayan et al. (2018) prepared a new dataset for the task of pure abstractive summarization. This dataset is not suitable for extractive methods. The authors collected news articles and their corresponding one-line new summary from BBC website. The statistics of the dataset are as follows: 204,045 training article-pairs, 11,332 tuning article pairs, and 11,334 test article pairs. The source document size is on average 430 tokens, and target summary size is on average 23 tokens, which leads to extreme summarization; hence, the name X-sum was given to this dataset. Models trained and developed with this dataset are evaluated using ROUGE (1, 2, L) scores. The dataset can be downloaded.^4^

#### 2.6.4 Sentence compression Google dataset

In 2013, Google developed a dataset for the task of sentence compression (Filippova and Altun, 2013), which may be considered as summarization at the sentence level. The first version of this dataset was released with 10*k* pairs; recently, Google released an updated version of this dataset which contains 200k pairs. The dataset can be downloaded from the link.^5^

### 2.7 Resources for text summarization and simplification

Beside from the available resources for summarization alone and simplification alone, in this section we discuss the availability of corpus for the combined task of summarization and simplification.

#### 2.7.1 PLOS and eLife datasets

Two datasets PLOS and eLife were introduced for the task of summarization and simplification, and both datasets focus on scientific documents from the biomedical domain and their corresponding summaries in plain English. The datasets were created by parsing XML articles in Python, the datasets are further structured into sections such as abstracts and article text, and article text is further formatted into subsections as per the headings presented in the original articles (Goldsack et al., 2022)

## 3 Data and method

Hybrid text simplification and summarization models require data in the form of a parallel corpus containing complex-simple pairs. To create such a corpus manually, we need domain knowledge and extensive time. We used the publicly available dataset from Zaman et al. (2020), and this dataset consists of 5,204 article and summary pairs. The corpus links full-text scientific articles and their abstracts to simplified summaries taken from EurekAlert.

### 3.1 Simplification aware text summarization (SATS)

We have so far described our baseline models, taken from the literature. Each of these models is designed for either simplification or summarization, except HTTS no one model is designed to simultaneously complete both tasks. HTSS is the only model that is a hybrid of summarization and simplification. To address the limitations of HTSS such as general language errors and grammar issues, we propose an adaptation to the ProphetNet architecture, which allows a summarization model to prioritize simple terms during generation. First, we need to understand what makes a word complex. While significant work has been done on automated prediction of lexical complexity (Shardlow et al., 2022), it is a well-established fact in the literature that lexical frequency is a strong indicator of how difficult a reader will find a word to understand (Paetzold and Specia, 2016; Martin et al., 2020a; North et al., 2023). As such, we take a corpus of 13,588,391 words, each associated with a frequency value which was derived as the result of counting word frequencies from over 1 trillion words of English web-text (Brants, 2006).

**Figure 1:**
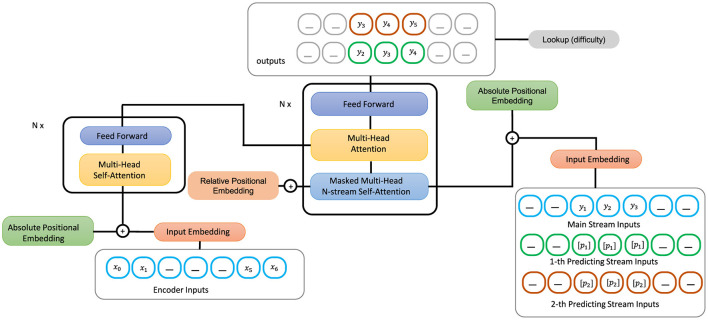
Proposed architecture of SATS model.

In our proposed model which is depicted in Figure 1, lookup-difficulty module is the contribution that we add to the summarization model to enhance its capability for the task of simplification. This module is responsible for guiding the generation of the underlying transformer model and ensuring that the generated output summary is simple to read by contributing values of difficulty scores to the loss function. The loss function is further used to update the parameters of the entire network.

We use this data and reformulate this idea into difficulty scores as in Equation (3):


(3) $$score_{log}=\frac{1}{log(w_{i})}$$

Here, *score*_*log*_ is the intermediate score which is further normalized between 0 and 1 as shown in Equation (4):


(4) $$score_{difficulty}=\frac{score_{log}-min(score_{log})}{max(score_{log})-min(score_{log})}$$

Here *score*_*difficulty*_ is the final difficulty score that we use for computing simplification. This score is in the range 0–1, and scales with the log of the frequency to avoid very common words overly influencing the simplification. We pre-compute *score*_*log*_, and *score*_*difficulty*_ for every word in our word frequency dataset and create a lookup table, which can be accessed during the generation phase.

We reformulate the difficulty score as a loss term in the following manner, relying on the lookup table to identify the difficulty score of the given token as demonstrated in Equation (5).


(5) $$L_{simp}=\frac{1}{T}\sum_{i=1}^{T}lookup_{score_{difficulty}}(w_{i})$$

In Equation (5), *T* is the size of the predictions output of ProphetNet. We updated the objective function by adding simplification loss from Equation (5), thus, the updated objective can be written as in shown in Equation (6).


(6) $$\begin{array}{l} \;L=\underset{LM_{loss}}{\underset{︸}{-\alpha_{0}\cdot (\sum_{t=1}^{T}\log P\theta (y_{t}|y_{<t},x))}}\\ \underset{Future\;n\text{-}gram_{loss}}{\underset{︸}{-\sum_{j=1}^{m-1}\alpha_{t}\cdot (\sum_{t=1}^{T-j}\log P\theta (y_{t+j}|y_{<t},x))}}\\ \;\underset{\text{simplification loss}}{\underset{︸}{-\lambda \cdot L_{simp}}} \end{array}$$

In Equation (6), *L*_*simp*_ is the simplification loss and λ is a hyperparameter used to control the degree of the effect of simplification loss as compared to the overall loss. Note that we tuned lambda in our experiments using the validation set and set it to 0.8. An example of the calculation of our simplification loss is shown in Table 2.

**Table 2:** Sample of words and their frequencies along with log score and simplification loss.

**Vocab Index**	**Word**	**Frequency**	***score*_*log*_**	***L*_*simp*_**
1	Where	282,489,721	0.80212354	0.05503001
2	Business	280,687,568	0.801758097	0.055149779
3	Must	269,659,445	0.799469364	0.055901677
⋮	⋮	⋮	⋮	⋮
5,725	Barometers	59,714	0.318946422	0.335138044
5,726	Scribing	59,712	0.318944509	0.335140006
5,727	Splattering	57,154	0.31644443	0.337714815
⋮	⋮	⋮	⋮	⋮
77,530	Antrum	37,668	0.292636921	0.363306085
77,531	Kudu	37,640	0.29259446	0.363353536
77,532	Victimizing	6,857	0.195363413	0.492969086

## 4 Results

For our experiments, we designed a computational environment using Python version 3.7 as a scripting language and PyTorch as a deep learning framework; in our experiments, we leveraged two datasets: (1) CNN-Daily mail specialized for text summarization and (2) Eureka Alert dataset specialized for hybrid text summarization and text simplification. For the evaluation of our proposed SATS model and comparison with baseline models, we used ROUGE as an automatic metric for text summarization, SARI and FKGL as an automatic metric for text simplification, BERTScore for semantic similarity and overall goodness of the generated output, and CSS as an automatic metric for combined text summarization and text simplification.

To generate the automatic evaluation scores for the baseline systems against the datasets that we used in our experiments, we retrained the implementation of three baseline systems and applied these to the Eureka dataset specialized for summarization and simplification. The first, HTSS, is a state-of-the-art joint simplification-summarization model. It is a hybrid model that implements a binary simplification loss to the pointer-generator architecture. The second is ACCESS, which provides controllable text simplification through a sequence-to-sequence architecture. The third is MUSS. Finally, we have implemented ProphetNet as described above and included the results with our adjusted loss function. The results are in Tables 3, 4.

**Table 3:** Compare SATS (proposed) with HTSS, ACCESS, MUSS, and ProphetNet using the Eureka Dataset.

**Model**	**Type**	**BERTscore**	**FKGL**	**ROUGE1**	**ROUGE2**	**ROUGEL**	**SARI**	**CSS_1_**
ACCESS (Martin et al., 2020a)	Simplification	82.34	7.52	9.641	1.177	7.893	38.3834	15.411
MUSS (Martin et al., 2020b)	Simplification	**84.15**	7.34	22.714	7.425	19.646	32.093204	26.60106
ProphetNet (Qi et al., 2020)	Summarization	83.42	7.25	33.414	9.443	18.962	40.44	36.5987
HTSS (Zaman et al., 2020)	Hybrid	81.73	8.42	21.938	03.21	17.171	37.650	27.7225
SATS	Hybrid	83.38	7.35	**34.24**	**10.15**	**20.21**	**40.83**	**37.24**

The bold values indicate the system with the highest performing score.

**Table 4:** Compare SATS with HTSS, ACCESS, MUSS, and ProphetNet using CNN-Daily Mail Dataset.

**Model**	**Type**	**ROUGE1**	**ROUGE2**	**ROUGEL**	**SARI**	**CSS_1_**
ACCESS (Martin et al., 2020a)	Simplification	15.557	2.787	11.668	34.3696	21.481
MUSS (Martin et al., 2020b)	Simplification	22.933	9.575	15.334	36.501	28.165
ProphetNet (Qi et al., 2020)	Summarization	**44.36**	21.262	30.723	42.033	43.167
HTSS (Zaman et al., 2020)	Hybrid	37.1011	15.91226	32.522	35.420429	36.24128
SATS	Hybrid	44.26	**21.48**	**30.93**	**43.40**	**43.82**

The bold values indicate the system with the highest performing score.

### 4.1 Baseline models

To compare and validate our method, some baseline methods are required, since our method is a hybrid of simplification and summarization, so we used baseline methods for simplification, summarization, and hybrid, for simplification we used two models ACCESS and MUSS, and we ran their code to reproduce the results, and for text summarization we ran ProphetNet model and reproduce the results, whereas, for hybrid model comparison we used HTSS. Details of each baseline method are given below.

#### 4.1.1 ACCESS

For text simplification, we choose ACCESS (Martin et al., 2020a) as a baseline. ACCESS is a text simplification model that uses extra control tokens to control the generated simplified text. ACCESS uses a sequence-to-sequence architecture which is based on the transformer model (Vaswani et al., 2017); furthermore, ACCESS is based on BART (Lewis et al., 2020).

#### 4.1.2 MUSS

MUSS (Martin et al., 2020b) is an unsupervised multilingual text simplification model that is based on ACCESS (Martin et al., 2020a) and BART (Lewis et al., 2020), from ACCESS it adapts the capability of controllable generation, and from BART, it adapts the sequence-to-sequence multilingual capability. MUSS uses mined sequences and paraphrasing to build the training dataset. There are two variations of MUSS, one is trained with mined sequences only. There is another version of MUSS which is supervised, this version is available for English only, and the supervised version uses the WikiLarge parallel corpus for training. We used the parallel version as a baseline for our study.

#### 4.1.3 HTSS

HTSS (Zaman et al., 2020) is a hybrid model for text simplification and text summarization. HTSS is based on the Pointer Generator model (See et al., 2017). We used HTSS implementation as it is without modification. To the best of our knowledge, HTSS is the only hybrid method for the combined task of summarization and simplification; thus, we consider it as a baseline model.

#### 4.1.4 ProphetNet

ProphetNet introduces an innovative pre-training model for sequence-to-sequence tasks, incorporating a unique self-supervised objective termed future n-gram prediction along with a newly proposed n-stream self-attention mechanism. In contrast to traditional sequence-to-sequence models that focus on one-step ahead prediction, ProphetNet optimizes for n-step ahead prediction. This entails predicting the next n tokens simultaneously based on the context tokens at each time step. This distinctive approach explicitly encourages the model to anticipate future tokens, thereby addressing concerns related to overfitting on strong local correlations. The model underwent pre-training using both a base-scale dataset (16GB) and an extensive large-scale dataset (160GB). Then, the model was fine-tuned for downstream tasks such as text summarization on CNN/Daily-Mail dataset and question answering on SQuAD 1.1 dataset.

### 4.2 Automatic evaluation

For the automatic evaluation of our proposed model and the baseline models, we used the ROUGE score as a metric for summarization SARI as a metric for simplification and CSS as a metric for combined summarization and simplification.

The results in terms of ROUGE scores SARI and CSS demonstrate that our newly proposed model: Simplification Aware Text summarization (SATS) significantly (*p* < 0.001 according to a paired *t*-test), outperforms ProphetNet on all metrics, although the gains in performance are reasonably small, with the majority of the improvement over other systems coming from the ProphetNet architecture itself. Our results demonstrate that ACCESS performs poorly on the summarization task showing that a simplification system alone is insufficient. ProphetNet gains a better SARI score than ACCESS (Martin et al., 2020a) and MUSS (Martin et al., 2020b), despite not being tuned for simplification. In our tuning, λ was set to 0.8, indicating its usefulness. Our model is better than all other models in terms of the Combined Simplification and Summarization metric (*CSS*_1_) score, which gives the harmonic mean of ROUGE1 and SARI, giving a new state of the art for the joint simplification and summarization task with our model.

We demonstrate that SATS outperforms ProphetNet and ACCESS on both a simplification dataset (Eureka) and a summarization dataset (CNN-Daily Mail). This demonstrates that the system we have developed is effective for the joint task of Simplification Aware Text Summarization that we set out to achieve. We also compared to the HTSS system, which is the former state of the art in terms of the CSS-1 score on the Eureka dataset, and we demonstrate that our new model outperforms HTSS not only on the Eureka Dataset but also on the CNN-Daily Mail dataset.

SATS is an adaptable architecture that can be fine-tuned for new datasets, and we envision that the results we have shown on the datasets we selected will be transferable to other datasets with the appropriate training being first undertaken. We have documented the model development and training process throughout our study to enable future researchers to replicate our results where needed. We also release our code and model checkpoints for future researchers to work with.

Further to the analysis that we have presented in Tables 3, 4, we also present examples of the best and worst summaries provided by our system (SATS) in Tables 5, 6. It is clear that when the system is performing well, the summaries are of a high quality and reflect the semantic content of the references, while not directly corresponding to the lexical and syntactic choices made by the original authors. The poor summaries are typically single lines that contain technical content. It is possible that this is emblematic of the model not being able to provide summaries for such content, which has led to a poor final output.

**Table 5:** Examples of good summaries generated by SATS.

**S. No**	**Reference**	**Generated**
1	“Women in Sub-Saharan Africa who use modern contraceptives are more likely to be tested for HIV than those who do not according to a study published April 25 2016 in the open-access journal Plos One by Katherine Center from the University of Arizona and colleagues. HIV/AIDS remains a major public health challenge for women in Sub-saharan Africa and medical appointments for use of modern contraceptives could provide an opportunity to offer confidential HIV testing”	“A new study published in Plos medicine finds that women who go to the doctor for contraception are more likely to be tested for HIV than those who use traditional methods of contraception according to a study published April 25 2016 in the open-access journal Plos One by Marie-Louise Derez from the University of Oxford UK and colleagues. HIV/AIDS is a major public health problem in sub-saharan Africa and the world health organization”
2	“A study published today in the journal Plos Medicine has identified the five genetic variants associated with higher levels of the branched-chain amino acids isoleucine leucine and valine. the researchers also found that these genetic variants were associated with an increased risk of type 2 diabetes. The researchers led by the medical research council (MRC) epidemiology unit at the University of Cambridge used large-scale genetic data together with detailed measurements of the branched-chain amino acids and”	“A study led by the max planck institute for the science of human genetics (LMU) in collaboration with colleagues in Germany and the united states has found that higher levels of the branched-chain amino acids isoleucine leucine and valine are associated with a higher risk of type 2 diabetes. The study was published in Plos Genetics. High levels of these amino acids have been linked to increased risk of diabetes in people”
3	“Polar bears, use of land during substantial summer sea ice loss in the Chukchi Sea increased by 30 days according to a study published November 18 2015 in the open-access journal Plos One by Karyn rode from the U.S. Geological Survey Alaska and colleagues. recent observations suggest that polar bears increasingly use land habitats where they have minimal access to their preferred prey likely in response to loss of their sea ice habitat associated with climatic warming. The authors of this study used location data”	“Polar bears are increasingly using land habitats in some parts of their range where they have minimal access to their preferred prey according to new research published in the open-access journal Plos one. Polar bears in the Chukchi Sea in Russia have increasingly used land habitats during the summer and in the winter according to a study published November 18 2015 in the peer-reviewed journal Plos One by Alexander Kravchenko from the Russian academy of sciences and colleagues in the summer sea-ice loss”

**Table 6:** Examples of bad summaries generated by SATS.

**S. No**	**Reference**	**Generated**
1	“Massachusetts general hospital (MGH) investigators may have uncovered a novel mechanism behind the ability of the diabetes drug Metformin to inhibit the progression of pancreatic cancer. In their report that has been published in the open access journal Plos One the research team describes finding that Metformin decreases the inflammation and fibrosis characteristic of the most common form of pancreatic cancer. Their findings in cellular and animal models and in patient tumor samples also indicate that this beneficial effect may be”	“Pancreatic ductal adenocarcinoma (PDAC) is a highly desmoplastic tumor with a dismal prognosis”
2	“Studies find that female meerkats can produce up to twice as much testosterone as their male counterparts. Their unusual hormone levels fuel their bullying behavior but at a cost photo courtesy of Charli Davies Duke University. Testosterone. It's often lauded as the hormone that makes males bigger bolder stronger. Now a pair of Duke University studies has identified one group of animals the meerkats of the southern tip of Africa in which females can produce>	< this is a female meerkat (Suricata Suricatta)”
3	“Taking human immune diversity into account increases the clinical relevance of immunological research. La Jolla institute for allergy and immunology La Jolla CA —mouse models have advanced our understanding of immune function and disease in many ways but they have failed to account for the natural diversity in human immune responses. As a result insights gained in the lab may be lost in translation. In their latest study researchers at La Jolla institute for allergy”	"This is a hybrid mouse diversity panel (HMDP) of 83 inbred strains of lipopolysaccharide (LPS)”

### 4.3 Human evaluation

**Figure 2:**
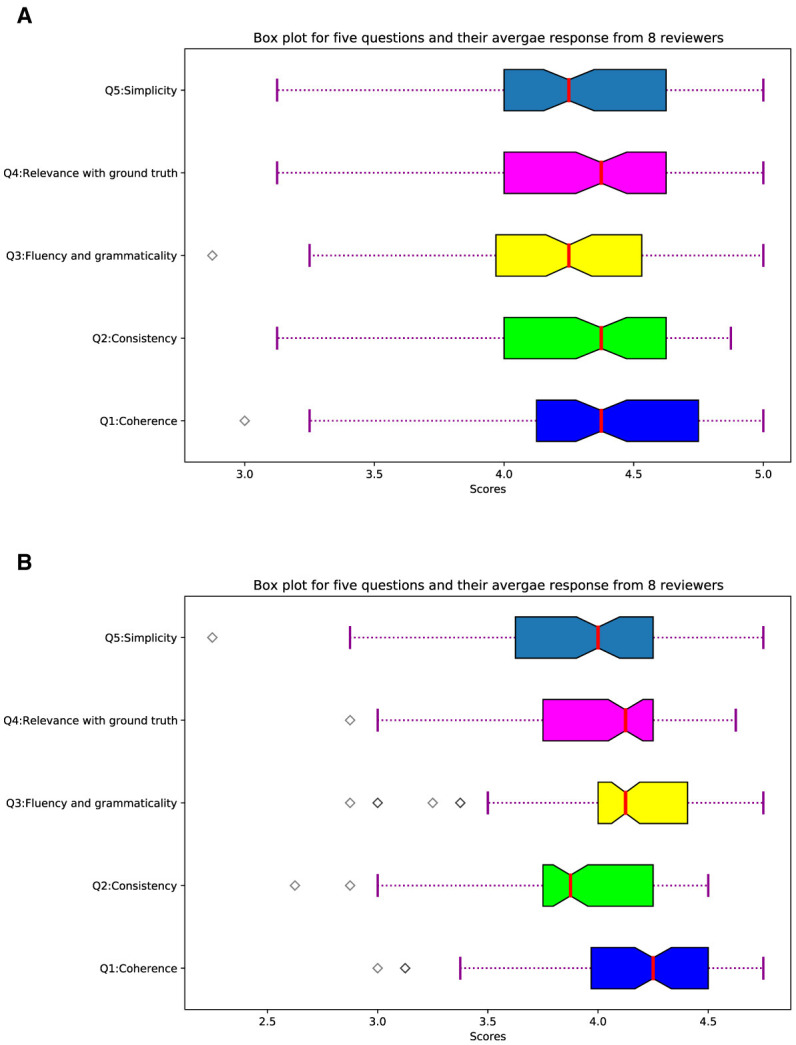
Box plot of five questions responses for 100 summaries. **(A)** SATS. **(B)** ProphetNet.

The quality of machine-generated summaries can be judged by automatic means such as ROUGE scores. These automatic metrics have their own limitations, such as the ROUGE metric favors short summaries and sometimes scores higher than expected in cases of extractive summarization. In the case of abstractive summarization, ROUGE score may be low for semantically identical summaries with high lexical differences. Therefore, we evaluated the summaries generated by SATS, and ProphetNet using a group of seven PhD students and one Masters student, all studying in computer science. We randomly selected 100 summaries from our test set. We then pose five questions about the quality of each summary following the study of Fabbri et al. (2021). Detail about these 5 questions is presented in Table 7, and we asked each question on a LIKERT scale from 1 to 5 where 5 means very good and 1 means very low. We carefully analyze responses from eight respondents and box plot responses from eight respondents and present it in Figure 2, and the mean in the box plot for each of our evaluation questions shows a positive ranking overall across 100 selected summaries. To investigate statistical significance, We further perform a Friedman test (Sawilowsky and Fahoome, 2005). We observed that statistic = 15.43 and *p* = 0.031, to interpret this *P*-value less than 0.05 means that the null hypothesis which states that the means of our observation are the same, can be rejected.

**Table 7:** Evaluation questions.

**S. No**	**Question**	**Explanation**
Q1: Coherence	How would you rate the Coherency of the Generated summary? On a scale from 1 to 5	The summary should be well-structured and well-organized. The summary should not just be a heap of related information, but should build from sentence to sentence to a coherent body of information about a topic; all the sentences should be well connected and have an overall theme or topic
Q2: Consistency	How would you rate the Consistency of the Generated summary? On a scale from 1 to 5	The factual alignment between the summary and the summarized source. A factually consistent summary contains only statements that are entailed in the source document. penalize summaries that contain hallucinated (non-existent) facts. A consistent summary should contain all the facts and the correct information.
Q3: Fluency and Grammatical	How would you rate the fluency and grammar of the Generated summary? On a scale from 1 to 5	The quality of individual sentences. Sentences in the summary should have no formatting problems, capitalization errors, or ungrammatical sentences (e.g., fragments, missing components) that make the text difficult to read. A fluent and grammatically correct summary should be easy to follow and have a natural flow in its sentences.
Q4: Relevance with ground truth	Is the generated summary Relevant to the ground truth summary? On a scale from 1 to 5	The generated Summary by the model is easy to understand for non-native speakers. And is relevant to the gold standard summary provided. the information and key idea presented in the summary should match the key idea of the gold standard summary.
Q5: Simplicity	Is the generated summary simple and easy to understand? On a scale from 1 to 5	Selection of important content from the source. The summary should include only important information from the source document. Penalize summaries that contain redundancies and excess information. the generated summary should be as easy to understand for a level of high school students.

**Figure 3:**
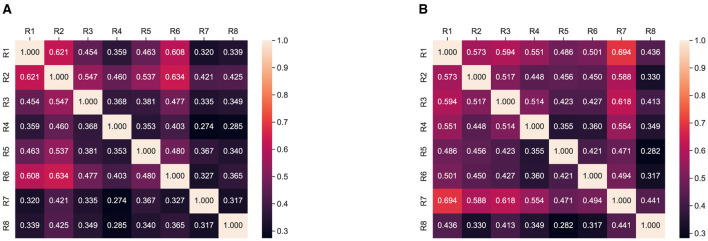
Overall summarization and simplification Interrater agreement: Cohen Kappa score between respondents. **(A)** SATS. (**B)** ProphetNet.

To further analyze the responses, we planned to compute inter-rater agreement using Cohen kappa score (Cohen, 1960; Artstein and Poesio, 2008); using this, we calculate the Cohen Kappa score for each question of one responded to each question of the rest of the *n*−1 responded and then average across five questions for each respondent; thus, we obtain an average score of five questions for each responded. Finally, we compute and present this analysis in Figure 3 where all possible agreements between the rater can be observed.

**Figure 4:**
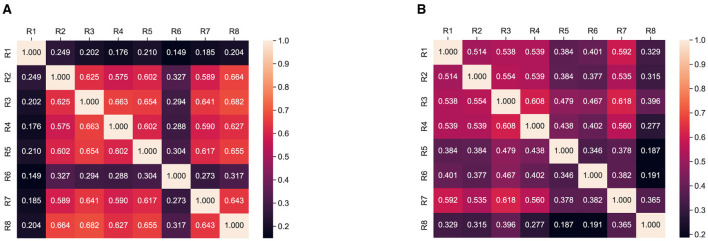
Interratter agreement: Cohen Kappa score for simplification between respondents. **(A)** SATS. **(B)** ProphetNet.

We investigated the simplification and summarization aspect simultaneously. In our human evaluation responses, we have a separate question for evaluating the easiness of our generated summaries. We analyzed the responses to question 5 and computed the Cohen Kappa score between the raters and presented our analysis in Figure 4. In Figure 4, we observe the majority of the raters show significant agreement that the generated summaries are simple and easy to understand.

## 5 Discussion

During our experiments and evaluation, we explored and found that summarization models perform better than the simplification models on the CNN-daily mail dataset. One of the possible reasons could be the structure and characteristics of the summarization dataset which is designed for the summarization task only. We also observed that summarization models perform better than the simplification model on the Eureka dataset. This is due to the easy word replacement of the simplification model which leads to generating different phrases as compared to the phrases present in the gold standard and thus leads to low scores as compared to the summarization task only. Our proposed SATS models improves the state-of-the-art HTSS model on the Eureka dataset and CNN-Daily Mail dataset. The improvement of the SATS model over ProphetNet is marginally low, this implies that adding a simplification module hurts the summarization capability of the model to some extent, the fact behind this is that summarization and simplification tasks often contradict each other, and such summarization aims at reducing the size of text and compressing while simplification aims at explaining and expanding for easiness in understanding. The CSS scores show overall improvements. In addition to this, the Rouge-L scores of the proposed SATS model are low on CNN-daily mail dataset as compared to prophetNet model due to the breaking of long phrases into simpler structures, and thus, long pieces could not match the gold set. Finally, human annotators found that SATS model produces a more simplified summary as compared to the ProphetNet model.

## 6 Concluding remarks

### 6.1 Limitations

During our experiments and evaluation, we observed that our proposed SATS model has some limitations such as it is unable to understand and interpret mathematical formulas presented in the given source document. Besides from this, the proposed model is also unable to interpret and incorporate information presented in the form of figures. Processing long input text is also an issue and needs to be addressed.

### 6.2 Future work

Efforts were made to propose a model that can combine the task of summarization with simplification and improve the previously established state of the art for the combined task, but the field of summarization and especially simplification requires more attention to address the limitation discussed in Section 6.1.

### 6.3 Conclusion

Our study has explored a new method of adapting ProphetNet or other loss-based sequence-to-sequence generation methods to produce simplified summaries of scholarly documents. We evaluated our summaries using automatic metrics and human judgments, and we found that our generated summaries are up to the mark. We have demonstrated that this leads to a significant improvement in the advancement of research in scientific communications. Our study shows that hybrid simplification and summarization are possible and that models can produce high-fidelity simplified summaries compared to reference texts. Future work incorporating larger model architectures and advances in simplification and summarization will doubtlessly lead to improvements on this helpful task in future - under the umbrella of science communication, that is, making science understandable for everyone.

## Data availability statement

Publicly available datasets were analyzed in this study. This data can be found at: https://github.com/slab-itu/HTSS/.

## Author contributions

FZ: Methodology, Writing – original draft, Writing – review & editing, Conceptualization, Data curation, Software. FK: Project administration, Supervision, Validation, Writing – original draft, Writing – review & editing. MS: Conceptualization, Data curation, Formal analysis, Investigation, Writing – original draft, Writing – review & editing. S-UH: Conceptualization, Investigation, Methodology, Supervision, Writing – original draft, Writing – review & editing. AK: Writing – original draft, Writing – review & editing. NA: Writing – original draft, Writing – review & editing.

## Footnotes

^1^ https://github.com/louismartin/dress-data/

^2^ https://github.com/facebookresearch/asset

^3^ https://github.com/abisee/cnn-dailymail

^4^ https://github.com/EdinburghNLP/XSum

^5^ https://github.com/google-research-datasets/sentence-compression

## References

[B1] Acharya S. Boyd A. D. Cameron R. Lopez K. D. Martyn-Nemeth P. Dickens C. . (2019). “Incorporating personalization features in a hospital-stay summary generation system,” in Proceedings of the 52nd Hawaii International Conference on System Sciences (Hawaii), 4175–4184. 10.24251/HICSS.2019.505

[B2] Agrawal S. Carpuat M. (2019). “Controlling text complexity in neural machine translation,” in Proceedings of the 2019 Conference on Empirical Methods in Natural Language Processing and the 9th International Joint Conference on Natural Language Processing (EMNLP-IJCNLP) (Hong Kong: Association for Computational Linguistics), 1549–1564. 10.18653/v1/D19-1166

[B3] Aharoni R. Goldberg Y. (2018). “Split and rephrase: better evaluation and stronger baselines,” in Proceedings of the 56th Annual Meeting of the Association for Computational Linguistics (Volume 2: Short Papers) (Melbourne, VIC: Association for Computational Linguistics), 719–724. 10.18653/v1/P18-2114

[B4] Al-Thanyyan S. S. Azmi A. M. (2021). Automated text simplification: a survey. ACM Comput. Surv. 54, 1–36. 10.1145/344269536083212

[B5] Alva-Manchego F. Martin L. Bordes A. Scarton C. Sagot B. Specia L. . (2020a). “ASSET: a dataset for tuning and evaluation of sentence simplification models with multiple rewriting transformations,” in Proceedings of the 58th Annual Meeting of the Association for Computational Linguistics (Association for Computational Linguistics), 4668–4679. 10.18653/v1/2020.acl-main.424

[B6] Alva-Manchego F. Martin L. Scarton C. Specia L. (2019). “EASSE: easier automatic sentence simplification evaluation,” in Proceedings of the 2019 Conference on Empirical Methods in Natural Language Processing and the 9th International Joint Conference on Natural Language Processing (EMNLP-IJCNLP): System Demonstrations (Hong Kong: Association for Computational Linguistics), 49–54. 10.18653/v1/D19-3009

[B7] Alva-Manchego F. Scarton C. Specia L. (2020b). Data-driven sentence simplification: survey and benchmark. Comput. Linguist. 46, 135–187. 10.1162/coli_a_00370

[B8] Artstein R. Poesio M. (2008). Inter-coder agreement for computational linguistics. Comput. Linguist. 34, 555–596. 10.1162/coli.07-034-R2

[B9] Azmi A. M. Altmami N. I. (2018). An abstractive arabic text summarizer with user controlled granularity. Inform. Process. Manag. 54, 903–921. 10.1016/j.ipm.2018.06.002

[B10] Banerjee S. Lavie A. (2005). “METEOR: an automatic metric for MT evaluation with improved correlation with human judgments,” in Proceedings of the ACL Workshop on Intrinsic and Extrinsic Evaluation Measures for Machine Translation and/or Summarization (Ann Arbor, MI: Association for Computational Linguistics), 65–72.

[B11] Barros C. Lloret E. Saquete E. Navarro-Colorado B. (2019). Natsum: narrative abstractive summarization through cross-document timeline generation. Inform. Process. Manag. 56, 1775–1793. 10.1016/j.ipm.2019.02.010

[B12] Baxendale P. B. (1958). Machine-made index for technical literature-an experiment. IBM J. Res. Dev. 2, 354–361. 10.1147/rd.24.035433813791

[B13] Botha J. A. Faruqui M. Alex J. Baldridge J. Das D. (2018). “Learning to split and rephrase from Wikipedia edit history,” in Proceedings of the 2018 Conference on Empirical Methods in Natural Language Processing (Brussels: Association for Computational Linguistics), 732–737. 10.18653/v1/D18-1080

[B14] Brants T. (2006). Web 1t 5-gram version 1. Available online at: https://catalog.ldc.upenn.edu/LDC2006T13 (accessed June 24, 2024).

[B15] Cai X. Shi K. Jiang Y. Yang L. Liu S. (2021). Hits-based attentional neural model for abstractive summarization. Knowl.-Based Syst. 222:106996. 10.1016/j.knosys.2021.106996

[B16] Carroll J. Minnen G. Canning Y. Devlin S. Tait J. (1998). “Practical simplification of english newspaper text to assist aphasic readers,” in Proc. of AAAI-98 Workshop on Integrating Artificial Intelligence and Assistive Technology (Madison, WI), 7–10.

[B17] Chen Y.-C. Bansal M. (2018). “Fast abstractive summarization with reinforce-selected sentence rewriting,” in Proceedings of the 56th Annual Meeting of the Association for Computational Linguistics (Volume 1: Long Papers) (Melbourne, VIC: Association for Computational Linguistics), 675–686. 10.18653/v1/P18-1063

[B18] Cho J. Seo M. Hajishirzi H. (2019). “Mixture content selection for diverse sequence generation,” in Proceedings of the 2019 Conference on Empirical Methods in Natural Language Processing and the 9th International Joint Conference on Natural Language Processing (EMNLP-IJCNLP) (Hong Kong), 3112–3122. 10.18653/v1/D19-1308

[B19] Cohen J. (1960). A coefficient of agreement for nominal scales. Educ. Psychol. Meas. 20, 37–46. 10.1177/001316446002000104

[B20] Collins E. Augenstein I. Riedel S. (2017). “A supervised approach to extractive summarisation of scientific papers,” in Proceedings of the 21st Conference on Computational Natural Language Learning (CoNLL 2017) (Vancouver, BC: Association for Computational Linguistics), 195–205. 10.18653/v1/K17-1021

[B21] Devlin J. Chang M.-W. Lee K. Toutanova K. (2019). “Bert: pre-training of deep bidirectional transformers for language understanding,” in Proceedings of the 2019 Conference of the North American Chapter of the Association for Computational Linguistics: Human Language Technologies, Volume 1 (Long and Short Papers) (Minneapolis, MN: Association for Computational Linguistics), 4171–4186.

[B22] Dong L. Yang N. Wang W. Wei F. Liu X. Wang Y. . (2019). “Unified language model pre-training for natural language understanding and generation,” in Advances in Neural Information Processing Systems (Vancouver, BC), 13042–13054.

[B23] Edmundson H. P. (1969). New methods in automatic extracting. J. ACM 16, 264–285. 10.1145/321510.321519

[B24] Fabbri A. R. Kryściński W. McCann B. Xiong C. Socher R. Radev D. (2021). Summeval: re-evaluating summarization evaluation. Trans. Assoc. Comput. Linguist. 9, 391–409. 10.1162/tacl_a_00373

[B25] Feng L. (2008). Text simplification: A survey. Technical Report. New York, NY: The City University of New York.

[B26] Filatova E. Hatzivassiloglou V. (2004). “Event-based extractive summarization,” in Text Summarization Branches Out (Barcelona: Association for Computational Linguistics), 104–111.

[B27] Filippova K. Alfonseca E. Colmenares C. A. Kaiser Ł. Vinyals O. (2015). “Sentence compression by deletion with lstms,” in Proceedings of the 2015 Conference on Empirical Methods in Natural Language Processing (Lisbon), 360–368. 10.18653/v1/D15-1042

[B28] Filippova K. Altun Y. (2013). “Overcoming the lack of parallel data in sentence compression,” in Proceedings of the 2013 Conference on Empirical Methods in Natural Language Processing (Seattle, WA), 1481–1491.

[B29] Giarelis N. Mastrokostas C. Karacapilidis N. (2023). Greekt5: a series of greek sequence-to-sequence models for news summarization. arXiv [Preprint]. arXiv:2311.07767. 10.48550/arXiv.2311.07767

[B30] Goldsack T. Zhang Z. Lin C. Scarton C. (2022). “Making science simple: corpora for the lay summarisation of scientific literature,” in Proceedings of the 2022 Conference on Empirical Methods in Natural Language Processing (Abu Dhabi), 10589–10604. 10.18653/v1/2022.emnlp-main.724

[B31] Goodfellow I. Pouget-Abadie J. Mirza M. Xu B. Warde-Farley D. Ozair S. . (2014). “Generative adversarial nets,” in Advances in neural information processing systems (Montreal, QC), 2672–2680.

[B32] Graff D. Kong J. Chen K. Maeda K. (2003). English gigaword, 4th Edn. Philadelphia, PA: Linguistic Data Consortium, 34.

[B33] Hoard J. E. Wojcik R. Holzhauser K. (1992). “An automated grammar and style checker for writers of simplified english,” in Computers *and Writing: State of the Art* (Dordrecht: Springer Netherlands), 278–296. 10.1007/978-94-011-2854-4_19

[B34] Hou J. Wang Y. Zhang Y. Wang D. (2022). How do scholars and non-scholars participate in dataset dissemination on twitter. J. Informetr. 16:101223. 10.1016/j.joi.2021.101223

[B35] Iqbal S. Hassan S.-U. Aljohani N. R. Alelyani S. Nawaz R. Bornmann L. . (2021). A decade of in-text citation analysis based on natural language processing and machine learning techniques: an overview of empirical studies. Scientometrics 126, 6551–6599. 10.1007/s11192-021-04055-1

[B36] Jia Q. Ren S. Liu Y. Zhu K. Q. (2023). Zero-shot faithfulness evaluation for text summarization with foundation language model. arXiv [Preprint]. arXiv:2310.11648. 10.48550/arXiv.2310.11648

[B37] Kamigaito H. Hayashi K. Hirao T. Nagata M. (2018). “Higher-order syntactic attention network for longer sentence compression,” in Proceedings of the 2018 Conference of the North American Chapter of the Association for Computational Linguistics: Human Language Technologies, Volume 1 (Long Papers) (New Orleans, LA: Association for Computational Linguistics), 1716–1726. 10.18653/v1/N18-1155

[B38] Kamigaito H. Okumura M. (2020). “Syntactically look-ahead attention network for sentence compression,” in Proceedings of the AAAI Conference on Artificial Intelligence, volume 34 (New York, NY), 8050–8057. 10.1609/aaai.v34i05.6315

[B39] Kincaid J. P. Fishburne Jr R. P. Rogers R. L. Chissom B. S. (1975). Derivation of new readability formulas (automated readability index, fog count and flesch reading ease formula) for navy enlisted personnel. Technical report. Millington, TN: Naval Technical Training Command. 10.21236/ADA006655

[B40] Kinugawa K. Tsuruoka Y. (2017). “A hierarchical neural extractive summarizer for academic papers,” in JSAI International Symposium on Artificial Intelligence (New York, NY: Springer), 339–354. 10.1007/978-3-319-93794-6_25

[B41] Lewis M. Liu Y. Goyal N. Ghazvininejad M. Mohamed A. Levy O. . (2020). “BART: denoising sequence-to-sequence pre-training for natural language generation, translation, and comprehension,” in Proceedings of the 58th Annual Meeting of the Association for Computational Linguistics (Association for Computational Linguistics), 7871–7880. 10.18653/v1/2020.acl-main.703

[B42] Li T. Li Y. Qiang J. Yuan Y.-H. (2018). “Text simplification with self-attention-based pointer-generator networks,” in Neural Information Processing, eds. L. Cheng, A. C. S. Leung, and S. Ozawa (Cham: Springer International Publishing), 537–545. 10.1007/978-3-030-04221-9_48

[B43] Lin C.-Y. (2004). “ROUGE: a package for automatic evaluation of summaries,” *in Text Summarization Branches Out* (Barcelona: Association for Computational Linguistics), 74–81.

[B44] Lin C.-Y. Hovy E. (2003). “Automatic evaluation of summaries using n-gram co-occurrence statistics,” in Proceedings of the 2003 Human Language Technology Conference of the North American Chapter of the Association for Computational Linguistics (Stroudsburg, PA: ACM), 150–157. 10.3115/1073445.1073465

[B45] Lin C.-Y. Och F. J. (2004). “Automatic evaluation of machine translation quality using longest common subsequence and skip-bigram statistics,” in Proceedings of the 42nd Annual Meeting of the Association for Computational Linguistics (ACL-04) (Barcelona: ACL), 605–612. 10.3115/1218955.1219032

[B46] Liu F. Flanigan J. Thomson S. Sadeh N. Smith N. A. (2015). “Toward abstractive summarization using semantic representations,” in Proceedings of the 2015 Conference of the North American Chapter of the Association for Computational Linguistics: Human Language Technologies (Denver, CO: Association for Computational Linguistics), 1077–1086. 10.3115/v1/N15-1114

[B47] Liu L. Lu Y. Yang M. Qu Q. Zhu J. Li H. . (2018). Generative adversarial network for abstractive text summarization. Proc. AAAI Conf. Artif. Intell. 32, 8109–8110. 10.1609/aaai.v32i1.1214132701451

[B48] Liu Y. Fabbri A. R. Chen J. Zhao Y. Han S. Joty S. . (2023). Benchmarking generation and evaluation capabilities of large language models for instruction controllable summarization. arXiv [Preprint]. arXiv:2311.09184. 10.48550/arXiv.2311.09184

[B49] Liu Y. Lapata M. (2019). “Text summarization with pretrained encoders,” in Proceedings of the 2019 Conference on Empirical Methods in Natural Language Processing and the 9th International Joint Conference on Natural Language Processing (EMNLP-IJCNLP) (Hong Kong), 3721–3731. 10.18653/v1/D19-1387

[B50] Luhn H. P. (1958). The automatic creation of literature abstracts. IBM J. Res. Dev. 2, 159–165. 10.1147/rd.22.015933813791

[B51] Macdonald I. Siddharthan A. (2016). “Summarising news stories for children,” in Proceedings of the 9th International Natural Language Generation conference (Edinburgh: Association for Computational Linguistics), 1–10. 10.18653/v1/W16-6601

[B52] Mackie S. McCreadie R. Macdonald C. Ounis I. (2014). “Comparing algorithms for microblog summarisation,” in Information Access Evaluation. Multilinguality, Multimodality, and Interaction, eds. E. Kanoulas, M. Lupu, P. Clough, M. Sanderson, M. Hall, A. Hanbury, et al.(Cham: Springer International Publishing), 153–159. 10.1007/978-3-319-11382-1_1529107976

[B53] Mao X. Huang S. Shen L. Li R. Yang H. (2021). Single document summarization using the information from documents with the same topic. Knowl.-Based Syst. 228:107265. 10.1016/j.knosys.2021.107265

[B54] Marchisio K. Guo J. Lai C.-I. Koehn P. (2019). “Controlling the reading level of machine translation output,” in Proceedings of Machine Translation Summit XVII Volume 1: Research Track (Dublin: European Association for Machine Translation), 193–203.

[B55] Martin L. de la Clergerie É. Sagot B. Bordes A. (2020a). “Controllable sentence simplification,” in Proceedings of the 12th Language Resources and Evaluation Conference (Marseille: European Language Resources Association), 4689–4698.

[B56] Martin L. Fan A. de la Clergerie É. Bordes A. Sagot B. (2020b). Multilingual unsupervised sentence simplification. arXiv [Preprint]. arXiv:2005.00352. 10.48550/arXiv.2005.00352

[B57] Mehta P. Majumder P. (2018). Effective aggregation of various summarization techniques. Inform. Process. Manag. 54, 145–158. 10.1016/j.ipm.2017.11.002

[B58] Mihalcea R. Tarau P. (2004). “TextRank: bringing order into text,” in Proceedings of the 2004 Conference on Empirical Methods in Natural Language Processing (Barcelona: Association for Computational Linguistics), 404–411.

[B59] Nallapati R. Zhou B. dos Santos C. Gulcehre C. Xiang B. (2016). “Abstractive text summarization using sequence-to-sequence RNNs and beyond,” in Proceedings of The 20th SIGNLL Conference on Computational Natural Language Learning (Berlin: Association for Computational Linguistics), 280–290. 10.18653/v1/K16-1028

[B60] Narayan S. Cohen S. B. Lapata M. (2018). “Don't give me the details, just the summary! topic-aware convolutional neural networks for extreme summarization,” in Proceedings of the 2018 Conference on Empirical Methods in Natural Language Processing (Brussels: Association for Computational Linguistics), 1797–1807. 10.18653/v1/D18-1206

[B61] Narayan S. Gardent C. Cohen S. B. Shimorina A. (2017). “Split and rephrase,” in Proceedings of the 2017 Conference on Empirical Methods in Natural Language Processing (Copenhagen: Association for Computational Linguistics), 606–616. 10.18653/v1/D17-1064

[B62] Nenkova A. Vanderwende L. (2005). The impact of frequency on summarization. Tech. Rep. MSR-TR-2005. Redmond, WA: Microsoft Research, 101.

[B63] Nishihara D. Kajiwara T. Arase Y. (2019). “Controllable text simplification with lexical constraint loss,” in Proceedings of the 57th Annual Meeting of the Association for Computational Linguistics: Student Research Workshop (Florence: Association for Computational Linguistics), 260–266. 10.18653/v1/P19-2036

[B64] Nisioi S. Štajner S. Ponzetto S. P. Dinu L. P. (2017). “Exploring neural text simplification models,” in Proceedings of the 55th Annual Meeting of the Association for Computational Linguistics (Volume 2: Short Papers) (Vancouver, BC: Association for Computational Linguistics), 85–91. 10.18653/v1/P17-2014

[B65] North K. Zampieri M. Shardlow M. (2023). Lexical complexity prediction: an overview. ACM Comput. Surv. 55, 1–42. 10.1145/355788521489393

[B66] Paetzold G. Specia L. (2016). “SemEval 2016 task 11: complex word identification,” in Proceedings of the 10th International Workshop on Semantic Evaluation (SemEval-2016) (San Diego, CA: Association for Computational Linguistics), 560–569. 10.18653/v1/S16-1085

[B67] Papineni K. Roukos S. Ward T. Zhu W.-J. (2002). “Bleu: a method for automatic evaluation of machine translation,” in Proceedings of the 40th Annual Meeting of the Association for Computational Linguistics (Philadelphia, PA: Association for Computational Linguistics), 311–318. 10.3115/1073083.1073135

[B68] Qi W. Yan Y. Gong Y. Liu D. Duan N. Chen J. . (2020). “ProphetNet: predicting future n-gram for sequence-to-SequencePre-training,” in Findings of the Association for Computational Linguistics: EMNLP 2020 (Association for Computational Linguistics), 2401–2410. 10.18653/v1/2020.findings-emnlp.217

[B69] Rehman T. Mandal R. Agarwal A. Sanyal D. K. (2023). Hallucination reduction in long input text summarization. arXiv [Preprint]. arXiv:2309.16781. 10.48550/arXiv.2309.16781

[B70] Sanchez-Gomez J. M. Vega-Rodríguez M. A. Perez C. J. (2020). A decomposition-based multi-objective optimization approach for extractive multi-document text summarization. Appl. Soft Comput. 91:106231. 10.1016/j.asoc.2020.106231

[B71] Sanchez-Gomez J. M. Vega-Rodríguez M. A. Pérez C. J. (2021). Sentiment-oriented query-focused text summarization addressed with a multi-objective optimization approach. Appl. Soft Comput. 113:107915. 10.1016/j.asoc.2021.107915

[B72] Sawilowsky S. Fahoome G. (2005). Friedman's test. Encycl. Stat. Behav. Sci. 10.1002/0470013192.bsa385

[B73] Schluter N. (2017). “The limits of automatic summarisation according to ROUGE,” in Proceedings of the 15th Conference of the European Chapter of the Association for Computational Linguistics: Volume 2, Short Papers (Valencia: Association for Computational Linguistics), 41–45. 10.18653/v1/E17-2007

[B74] See A. Liu P. J. Manning C. D. (2017). “Get to the point: summarization with pointer-generator networks,” in Proceedings of the 55th Annual Meeting of the Association for Computational Linguistics (Volume 1: Long Papers) (Vancouver: Association for Computational Linguistics), 1073–1083. 10.18653/v1/P17-1099

[B75] Shardlow M. (2014a). “Out in the open: finding and categorising errors in the lexical simplification pipeline,” in Proceedings of the Ninth International Conference on Language Resources and Evaluation (LREC-2014) (Reykjavik: European Languages Resources Association), 1583–1590.

[B76] Shardlow M. (2014b). A survey of automated text simplification. Int. J. Adv. Comput. Sci. Appl. 4, 58–70. 10.14569/SpecialIssue.2014.04010936083212

[B77] Shardlow M. Batista-Navarro R. Thompson P. Nawaz R. McNaught J. Ananiadou S. . (2018). Identification of research hypotheses and new knowledge from scientific literature. BMC Med. Inform. Decis. Mak. 18:46. 10.1186/s12911-018-0639-1 29940927 PMC6019216

[B78] Shardlow M. Evans R. Zampieri M. (2022). Predicting lexical complexity in english texts: the complex 2.0 dataset. Lang. Resour. Eval. 56, 1153–1194. 10.1007/s10579-022-09588-2

[B79] Siddharthan A. (2014). A survey of research on text simplification. ITL-Int. J. Appl. Linguist. 165, 259–298. 10.1075/itl.165.2.06sid33486653

[B80] Suleiman D. Awajan A. (2022). Multilayer encoder and single-layer decoder for abstractive arabic text summarization. Knowl.-Based Syst. 237:107791. 10.1016/j.knosys.2021.107791

[B81] Sulem E. Abend O. Rappoport A. (2018). “Semantic structural evaluation for text simplification,” in Proceedings of the 2018 Conference of the North American Chapter of the Association for Computational Linguistics: Human Language Technologies, Volume 1 (Long Papers) (New Orleans, LA: Association for Computational Linguistics), 685–696. 10.18653/v1/N18-1063

[B82] Thompson P. Nawaz R. McNaught J. Ananiadou S. (2017). Enriching news events with meta-knowledge information. Lang. Resour. Eval. 51, 409–438. 10.1007/s10579-016-9344-9

[B83] Tomer M. Kumar M. (2022). “STV-BEATS: skip thought vector and bi-encoder based automatic text summarizer. Knowl.-Based Syst. 240:108108. 10.1016/j.knosys.2021.108108

[B84] Van Veen D. Van Uden C. Blankemeier L. Delbrouck J.-B. Aali A. Bluethgen C. . (2023). Clinical text summarization: adapting large language models can outperform human experts. arXiv [Preprint]. arXiv:2309,07430. 10.48550/arXiv.2309.07430 PMC11479659 38413730

[B85] Vaswani A. Shazeer N. Parmar N. Uszkoreit J. Jones L. Gomez A. N. . (2017). “Attention is all you need,” in Advances in neural information processing systems (Long Beach, CA), 5998–6008.

[B86] Vinyals O. Fortunato M. Jaitly N. (2015). “Pointer networks,” in Advances in Neural Information Processing Systems, pages (Montreal, QC), 2692–2700.

[B87] Wang L. Jiang J. Chieu H. L. Ong C. H. Song D. Liao L. . (2017). “Can syntax help? improving an LSTM-based sentence compression model for new domains,” in Proceedings of the 55th Annual Meeting of the Association for Computational Linguistics (Volume 1: Long Papers) (Vancouver, BC: Association for Computational Linguistics), 1385–1393. 10.18653/v1/P17-1127

[B88] Wubben S. van den Bosch A. Krahmer E. (2012). “Sentence simplification by monolingual machine translation,” in Proceedings of the 50th Annual Meeting of the Association for Computational Linguistics (Volume 1: Long Papers) (Jeju Island: Association for Computational Linguistics), 1015–1024.

[B89] Xu W. Napoles C. Pavlick E. Chen Q. Callison-Burch C. (2016). Optimizing statistical machine translation for text simplification. Trans. Assoc. Comput. Linguist. 4, 401–415. 10.1162/tacl_a_00107

[B90] Yan Y. Qi W. Gong Y. Liu D. Duan N. Chen J. . (2020). Prophetnet: predicting future n-gram for sequence-to-sequence pre-training. arXiv [Preprint] arXiv:2001.04063. 10.48550/arXiv.2001.04063

[B91] Yang R. Zeng Q. You K. Qiao Y. Huang L. Hsieh C.-C. . (2023). Medgen: a python natural language processing toolkit for medical text processing. arXiv [Preprint]. arXiv:2311.16588. 10.48550/arXiv.2311.16588 PMC11487205 39361955

[B92] Zaman F. Shardlow M. Hassan S.-U. Aljohani N. R. Nawaz R. (2020). HTSS: a novel hybrid text summarisation and simplification architecture. Inform. Process. Manag. 57:102351. 10.1016/j.ipm.2020.102351

[B93] Zerva C. Nghiem M.-Q. Nguyen N. T. Ananiadou S. (2020). Cited text span identification for scientific summarisation using pre-trained encoders. Scientometrics 125, 3109–3137. 10.1007/s11192-020-03455-z

[B94] Zhang J. Zhao Y. Saleh M. Liu P. J. (2019a). Pegasus: pre-training with extracted gap-sentences for abstractive summarization. arXiv [Preprint]. arXiv:1912.08777. 10.48550/arXiv.1912.08777

[B95] Zhang T. Kishore V. Wu F. Weinberger K. Q. Artzi Y. (2019b). “Bertscore: evaluating text generation with Bert,” in International Conference on Learning Representations (New Orleans, LA).

[B96] Zhang X. Lapata M. (2017). “Sentence simplification with deep reinforcement learning,” in Proceedings of the 2017 Conference on Empirical Methods in Natural Language Processing (Copenhagen), 584–594. 10.18653/v1/D17-1062

[B97] Zhao Y. Luo Z. Aizawa A. (2018). “A language model based evaluator for sentence compression,” in Proceedings of the 56th Annual Meeting of the Association for Computational Linguistics (Volume 2: Short Papers) (Melbourne, VIC: Association for Computational Linguistics), 170–175. 10.18653/v1/P18-2028

[B98] Zhu Z. Bernhard D. Gurevych I. (2010). “A monolingual tree-based translation model for sentence simplification,” in Proceedings of the 23rd International Conference on Computational Linguistics (Coling 2010) (Beijing: Coling 2010 Organizing Committee), 1353–1361.

[B99] Zopf M. Loza Mencía E. Fürnkranz J. (2018). “Which scores to predict in sentence regression for text summarization?” in Proceedings of the 2018 Conference of the North American Chapter of the Association for Computational Linguistics: Human Language Technologies, Volume 1 (Long Papers) (New Orleans, LA: Association for Computational Linguistics), 1782–1791. 10.18653/v1/N18-1161

